# Supplementary material for: Current-induced magnetization switching in atom-thick tungsten engineered perpendicular magnetic tunnel junctions with large tunnel magnetoresistance
Source: Nat Commun. 2018 Feb 14;9:671. doi: 10.1038/s41467-018-03140-z (PMC5813193; doi:10.1038/s41467-018-03140-z)
Supplement: Supplementary file 1 — Supplementary Information [file 41467_2018_3140_MOESM1_ESM.docx]

**Supplementary Note 1. Magnetoresistance ratio and resistance area product comparison**

**
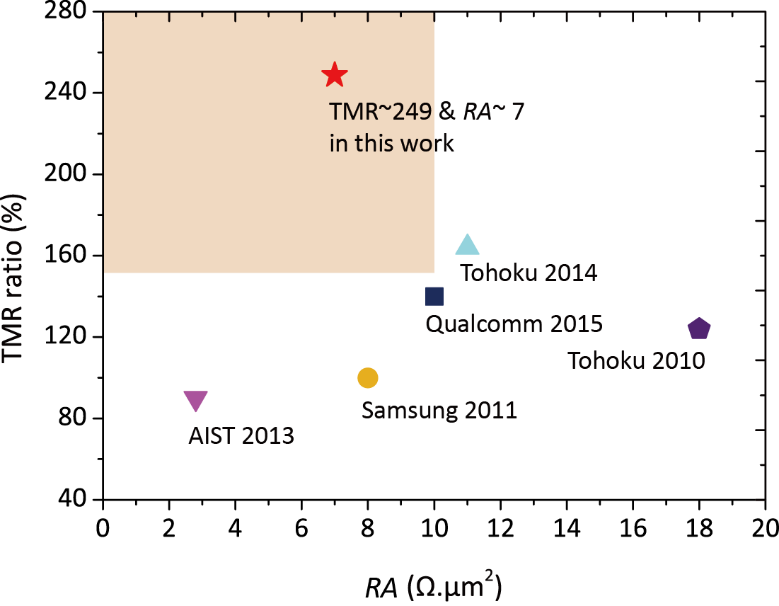
**

**Supplementary Figure 1 | TMR and *RA* comparison.** Data for p-MTJs that have been published with STT switching. The star shows the trade-off that have been achieved in our work. Background colour indicates the considerations to realize high-reliable STT-MRAM.^1^

To realize high-density and low-voltage operation for spin-transfer torque magnetic random access memories (STT-MRAMs), thin MgO layer is demanded to reduce resistance area product (*RA* < 10 Ω·µm^2^), whereas large tunnel magnetoresistance ratio (TMR > 150%) requires thick MgO layer for strong output signal.^1^ As mentioned in the main text, though the double MgO/CoFeB interfaces enable significant thermal stability factor (Δ) enhancement, the introduction of an additional MgO layer makes it even more difficult to achieve a tradeoff. For the convenience of comparison, we plotted those data that have been previously published in Supplementary Fig. 1. Among them, Tohoku University proposed the first perpendicular magnetic tunnel junction (p-MTJ) based on MgO/CoFeB/Ta structure in 2010,^2^ followed by its meaningful results measured from MgO/CoFeB/Ta/CoFeB/MgO free layer in 2014.^3^ Qualcomm Technologies also developed the similar configuration,^4^ while National Institute of Advanced Industrial Science and Technology (AIST) replaced CoFeB with FeB layers,^5^ and Samsung Electronic Corp used the classic design.^6^

Here, we report the current-induced magnetization switching in p-MTJs with W spacer and bridging layers, which at the same time exhibit a TMR as large as 249% and an *RA* around 7 Ω·µm^2^. It should be mentioned that though large TMRs have been found in the p-MTJ films similar to ours, no nano-pillar devices or STT switching have been presented currently for our collection.^7-8^ Besides, Ref. 9 reported a high TMR up to 215% and 180% for a p-MTJ device with STT switching, while no RA has been disclosed so far.

**Supplementary Note 2. Magnetic tunnel junction film optimization**

**
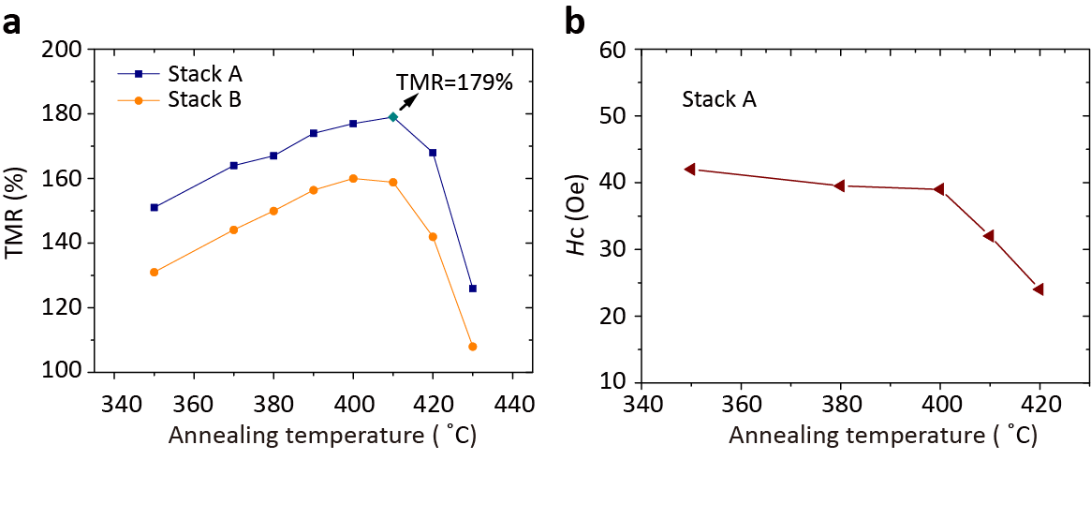
**

**Supplementary Figure 2 | TMR and *H*_C_ optimization. (a)** Current in-plane tunnelling (CIPT) results of TMR measured from stack A and B. All samples were subject to vacuum annealing from 350 °C to 430 °C for an hour after deposition. The triangle indicates the highest TMR (179%) obtained from stack B annealed at 410 °C. **(b)** *H*_C_ of stack B as a function of increasing annealing temperature.

The configuration of our p-MTJ film used for nanopillar fabrication has been carefully tuned, including the material and thickness of each layers. Here, we give some examples to explain our considerations.

In Supplementary Fig. 2a, the comparison of TMRs between p-MTJ films with MgO (0.8)/ CoFeB (1.3, 1.2)/ W (0.2, 0.3)/ CoFeB (0.5)/ MgO (0.75) (hereafter called stack A and B, numbers in parenthesis denote layer thickness in nm) free layers is shown with the change of vacuum annealing temperatures between 350 °C and 430 °C without magnetic field. The measurements were done with current in-plane tunnelling (CIPT) method. For stack A, the TMRs are about 20% larger than that of stack B, hence stack A is more favourable regarding TMR.

On the other hand, a large TMR of 179% was determined for stack A under 410 °C, although it started to drop at 420 °C. This significant enhancement of TMR and thermal endurance is related to the lower atom diffusion when using W, instead of Ta, as the spacer and bridging layers. As a result, annealing at higher temperature allows the improvement for the crystalline quality of the MgO barrier and the bcc texture of the CoFeB layers, ^2,10^ leading to a larger TMR. While drastic atom diffusion happened with further rising temperature, thus the TMR degraded above 410 °C annealing.

Supplementary Fig. 2b presents the coercivity (*H*_C_) dependence of stack B on annealing temperatures. It remains almost constant, 40 Oe, between 350 °C and 400 °C, then starts to degrade. Synthetically considering those factors, we chose stack B annealed at 390 °C for the study of nanopillars.

**Supplementary Note 3. Thermal stability factor estimation**

Because Δ is proportional to anisotropy field (*H_k_*), we measured *H_k_* by ferromagnetic resonance (FMR) method to determine the Δ of p-MTJ films with various bottom CoFeB thicknesses (*t*) in the MgO/CoFeB (*t* = 1.2~1.6)/ W (0.2)/ CoFeB (0.5)/ MgO free layer. *H_k_* was found to decrease from 4313 Oe to -663 Oe, indicating a transition from strong perpendicular magnetic anisotropy to in-plane magnetic anisotropy with increasing *t*. In addition, Supplementary Fig. 3 gives an example of the M-H hysteresis loops for *t* = 1.2 nm, where an *H*_C_ of 70 Oe and a shift field around 0 can be read from the minor loop.


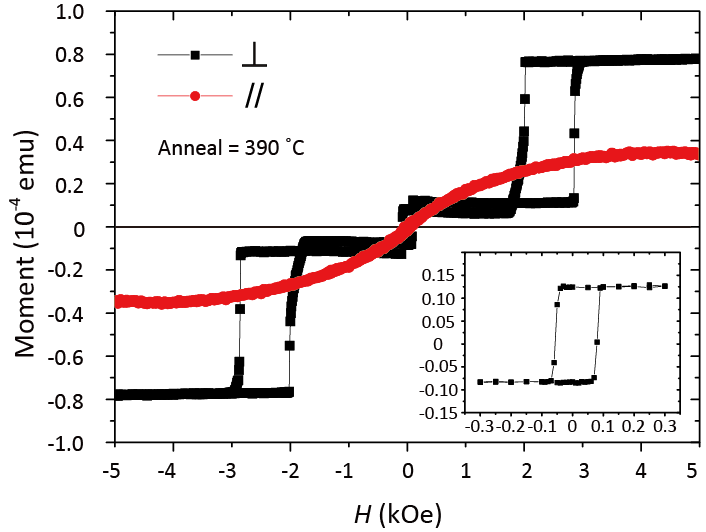


**Supplementary Figure 3 | Hysteresis loops measurement.** Out-of-plane ($\perp$) and in-plane ($\parallel$) magnetic fields induced hysteresis loops of the p-MTJ film annealed at 390 °C, where *t* = 1.2 nm; inset is the minor loop.

**Supplementary Note 4. Spin transfer torque switching at room temperature**

Nanopillars that we patterned from the p-MTJ film annealed at 390 °C have radius (*r*) from 45 nm to 150 nm. As shown in Supplementary Fig. 4, the p-MTJ with *r* = 45 nm demonstrates a critical current density *J*_C_ = +2.2/-3.4 MA∙cm^-2^. The values of *RA* calculated from devices with various dimensions are all around 7 Ω·µm^2^, we thus can conclude that this lower value is not caused by current shunting.


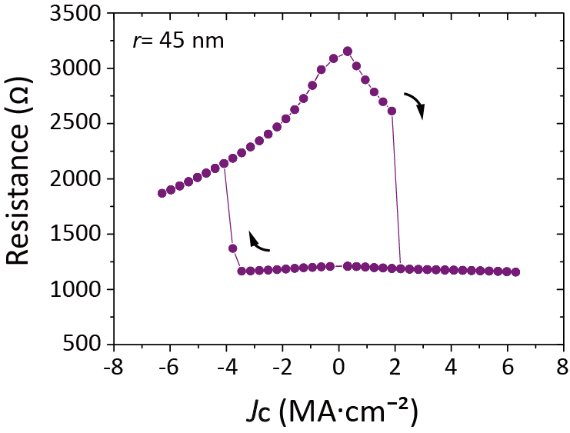


**Supplementary Figure 4 | STT measurements at room temperature.** Magnetoresistance versus DC current curves measured from p-MTJs with *r* = 45 nm, and. Arrows denote the magnetization transitions from anti-parallel (AP) to parallel (P) states or the opposite situation.

**Supplementary Note 5. Spin transfer torque switching at low temperature**

We also characterized the STT switching at low temperature (35 K). Supplementary Fig. 5a and 5b show the representative resistance versus pulse current curves at 100 µs and 1 ms durations, respectively. Supplementary Fig. 5c presents *J*_C_ as a function of *τ*_P_*/τ*_0_, where *τ*_P_ is the pulse duration, and *τ*_0_ = 1 ns is the characteristic attempt time. It is easy to understand that without the assistance of thermal fluctuation, the intrinsic critical current density *J*_C0_ increases to from 7.8 MA∙cm^-2^ to 8.5 MA∙cm^-2^.


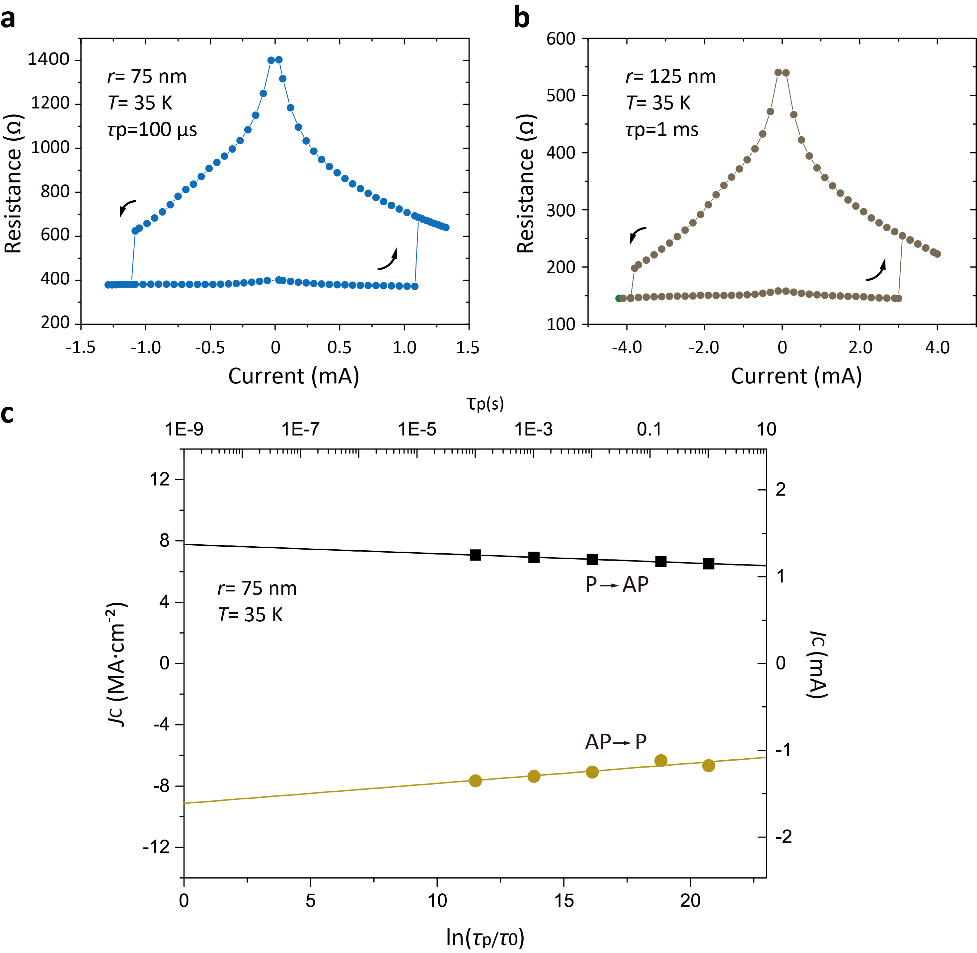


**Supplementary Figure 5 | STT measurements after optimization at low temperature.** Magnetoresistance versus pulse current curves measured from p-MTJs with **(a)** *r*=75 nm, and **(b)** *r*=125 nm at 100 µs and 1 ms durations. **(c)** *J*_C_ as a function of ln(*τ*_P_*/τ*_0_) measured from p-MTJ with *r*=75 nm. Arrows denote the magnetization transitions from AP to parallel P states or the opposite situation.

**Supplementary Note 6. Calculation set up**

The atomic structures used for our first-principles calculation is Ta (001)/ CoFe (001)/ X/ CoFe (001)/ MgO (001)/ CoFe (001)/ Ta (001), where the X represents W or Ta spacer layers (Supplementary Fig. 6), and Ta was chosen as the material of the semi-infinite electrodes. The charge transport in the p-MTJ layers was along the z direction, while the x and y lattice constant were fixed as 2.83 Å. We set X (001) [110] || CoFe (001) [100] as the crystallographic orientation of the X/CoFe interfaces to minimize the lattice mismatch (< 5%).^11^ A 20×20×1 k-point mesh was applied for the NEGF-DFT self-consistent calculation, and a much denser k-point sampling of 300×300×1 was used in the conductance and transmission coefficient analyses. By using the calculated conductance, TMR can be obtained with: $TMR=\frac{G_{P}-G_{\mathrm{AP}}}{G_{\mathrm{AP}}}$, where the *G*_P_ and *G*_AP_ denote the total conductance for parallel (P) and anti-parallel (AP) states, respectively. Note that we modelled the experimental p-MTJ configuration to the best of our abilities, except that the top MgO layer was omitted because it does not determine the spin transport in p-MTJ layers.


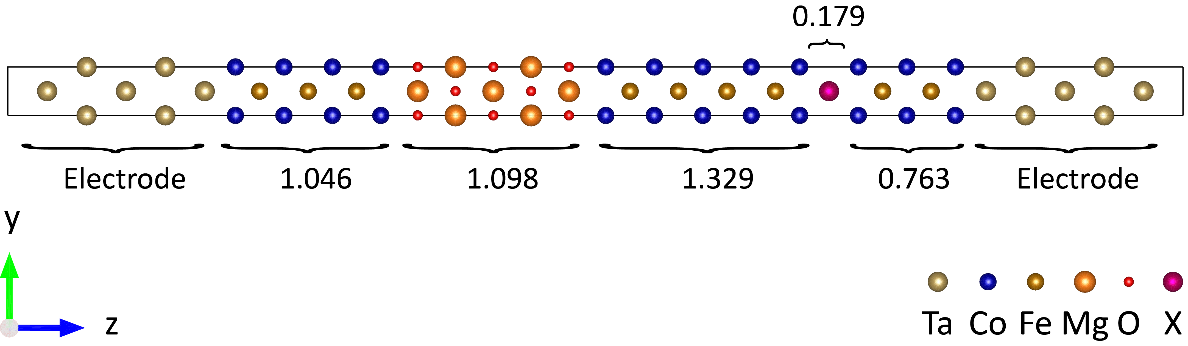


**Supplementary Figure 6 | Atomic structure for calculation.** Detail information for the atomic structures Ta/CoFe/X/CoFe/MgO/CoFe/Ta, where X represents W or Ta spacer layers. Numbers show the thickness of each layer in nm.

**Supplementary Note 7. Density of scattering states analyses**

The transmission coefficient in p-MTJ atomic structures could be analysed by the scattering states, which is the absolute square of the tunnelling electron wave function. Supplementary Fig. 7 illustrates the ***k****_||_*-resolved density of scattering states (DOSS) projected on Co atoms at CoFe/X interface at Fermi level *E_F_*. For the minority-spin condition in Supplementary Fig. 7b, f, sharp peaks of the DOSS appear at the region around ***k****_||_* = (0, 1) point (see in red circles), where high transmission coefficients occur, referring to Fig. 4 in the main text. This phenomenon suggests that resonant tunnelling transmission is proportional to the scattering state of interfacial Co atoms, while the scattering states are affected by the material of X spacer layer. In the case of W spacer layer, higher DOSS appears as shown in Supplementary Fig. 7b, leading to higher transmission coefficients in P state and higher TMR consequently.


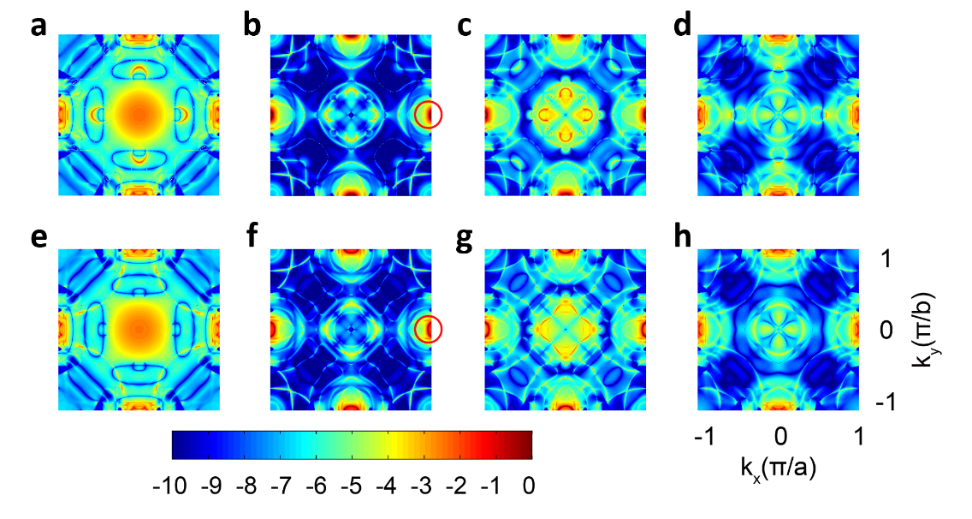


**Supplementary Figure 7 | Spin- and *k_||_*- resolved DOSS.** DOSS at *E_F_* for p-MTJ stacks with **(a)-(d)** W, and **(e)-(h)** Ta spacer layers. **(a) (e)** are the majority-to-majority conditions, and **(b) (f)** the minority-to-minority conditions in P state; **(c) (g)** are the majority-to-minority conditions, and **(d) (h)** the minority-to-majority conditions in AP state. The bar shows the DOSS from low (blue) to high (red).

**Supplementary Note 8. Atom diffusion**

We studied the electron energy-loss spectroscopy (EELS) of the core structure in the p-MTJ films annealed at 370 °C and 410 °C, *i.e.*, (0.25) W/ CoFeB (1.0)/ MgO (0.8)/ CoFeB (1.3)/ W (0.2)/ CoFeB (0.5)/ MgO (0.75). As mapped in Supplementary Fig. 8, B, Co, Fe, W, Mg, and O shows no further diffusion under increasing annealing temperatures, which allows higher TMR and thermal endurance.

**
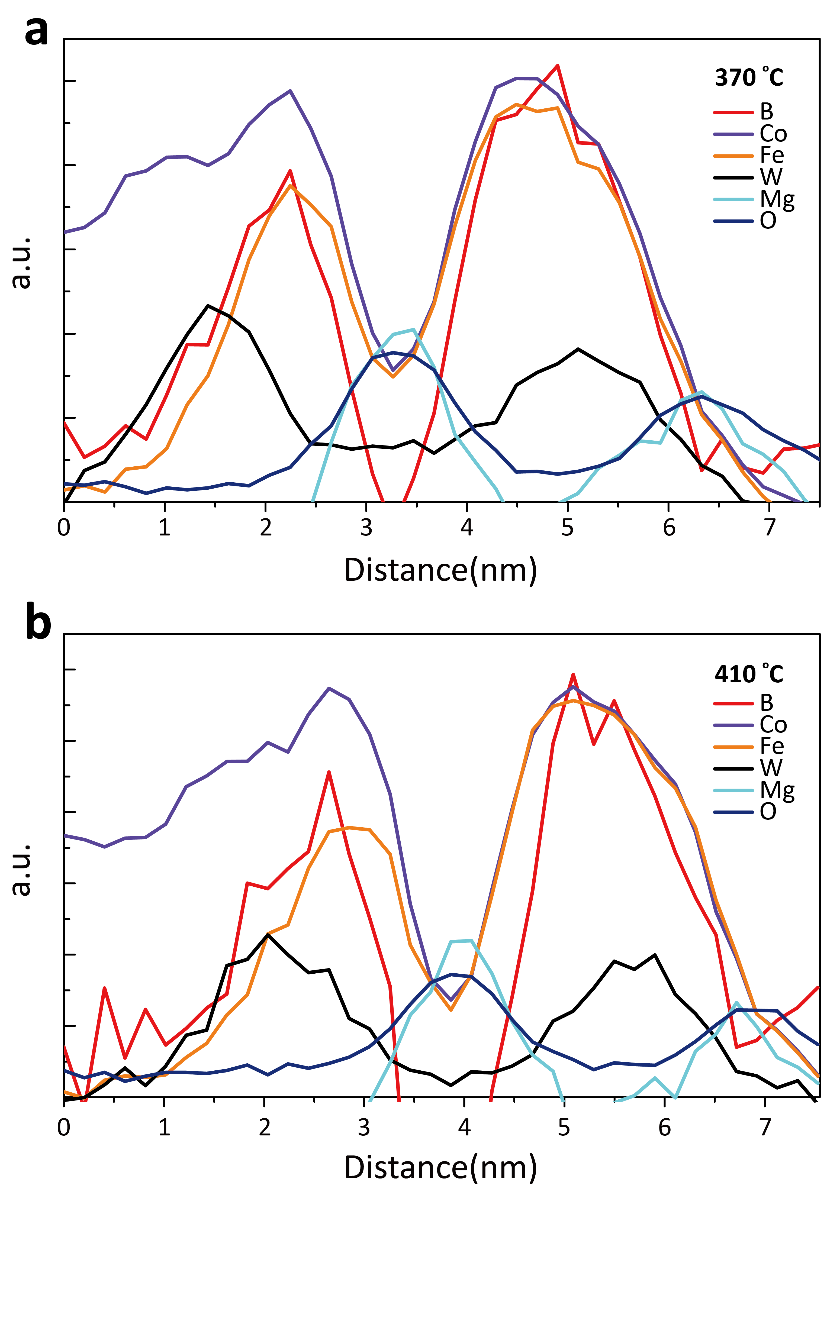
**

**Supplementary Figure 8 | EELS intensity profile of the core structure.** Atom distribution for samples annealed at **(a)** 370 °C, and **(b)** 410 °C.

**Supplementary Note 9. TMR and film thickness calculations**

We further established the first-principles calculations to present the relationship between TMR and film thickness of our devices more clearly.

First, we investigate TMR in terms of the W spacer layer thickness. The atomic structures were built according to our experimental p-MTJ configuration, including 7 CoFe/5 MgO/9 CoFe/1, 3 W/5 CoFe. As shown in Supplementary Table 1, the calculated TMR decreases from 245% to 162% while increasing the thickness of W spacer layer from one single atom to three (0.58 nm). Then, we modelled the p-MTJ stack with thicker upper and bottom CoFeB free layers, *i.e.*, 7 CoFe/5 MgO/11 CoFe/3 W/7 CoFe. It revealed that for the majority spin in P state, the spin-resolved conductance increased with enhanced coherent tunnelling. A TMR as high as 348% can be obtained by using thicker CoFeB free layers. Thus, the TMR depends on the two CoFeB free layers, W spacer layer, and MgO barriers, making it difficult to determine the configuration possessing the largest TMR. However, our main objective is to realize ultrahigh TMR with STT switching, thereby different device structure was investigated in our experiments to achieve this phenomenon.

**Supplementary Table 1 |** Spin-resolved conductance and TMR values in 7 CoFe/5 MgO/CoFe/W(Ta)/CoFe structures with various film thicknesses. The conductance unit is *e^2^/h*.

| MTJ film | Conductance (P) | Conductance (AP) | TMR |
| --- | --- | --- | --- |
| 9 CoFe/1 W/5 CoFe | 5.59×10^-5^ | 1.62×10^-5^ | 245% |
| 9 CoFe/1 Ta/5 CoFe | 3.92×10^-5^ | 2.07×10^-5^ | 89% |
| 9 CoFe/3 W/5 CoFe | 3.13×10^-5^ | 1.20×10^-5^ | 162% |
| 11 CoFe/3 W/7 CoFe | 6.10×10^-5^ | 1.36×10^-5^ | 348% |

**Supplementary References**

1. Zhao, W. S. et al. Design considerations and strategies for high-reliable STT-MRAM. *Microelectron. Reliab*. **51,** 1454-1458 (2011).
2. Ikeda, S. et al. A perpendicular-anisotropy CoFeB/MgO magnetic tunnel junction. *Nat.* *Mater.* **9,** 721–724 (2010).
3. Ikeda, S. et al. Perpendicular-anisotropy CoFeB-MgO based magnetic tunnel junctions scaling down to 1X nm. In *Electron Devices Meeting (IEDM), 2014 IEEE International*. **33-2,** (2014).
4. Park, C. et al. Systematic optimization of 1 Gbit perpendicular magnetic tunnel junction arrays for 28 nm embedded STT-MRAM and beyond. In *Electron Devices Meeting (IEDM), 2015 IEEE International*. **26-2,** (2015).
5. Yakushiji, K. et al. Ultralow-voltage spin-transfer switching in perpendicularly magnetized magnetic tunnel junctions with synthetic antiferromagnetic reference layer. *Appl. Phys. Express* **6**, 113006 (2013).
6. Kim, W. et al. Extended scalability of perpendicular STT-MRAM towards sub-20nm MTJ node. In *Electron Devices Meeting (IEDM), 2011 IEEE International*. **24-1,** (2011).
7. Lee, S. E., Shim, T. H., & Park, J. G. Perpendicular magnetic tunnel junction (p-MTJ) spin-valves designed with a top Co_2_Fe_6_B_2_ free layer and a nanoscale-thick tungsten bridging and capping layer. *NPG Asia Mater.* **9,** e324 (2016).
8. Tezuka, N. *et al.* Perpendicular magnetic tunnel junctions with low resistance-area product: high output voltage and bias dependence of magnetoresistance. IEEE Magn. Lett. 7, 3104204 (2016).
9. Song, Y. J. et al. Highly functional and reliable 8Mb STT-MRAM embedded in 28nm logic. In *Electron Devices Meeting (IEDM), 2016 IEEE International*. **27-2,** (2016).
10. Ikeda, S. *et al.* Tunnel magnetoresistance of 604% at 300 K by suppression of Ta diffusion in CoFeB/MgO/CoFeB pseudo-spin-valves annealed at high temperature. *Appl. Phys. Lett.* **93,** 082508 (2008).
11. Häglund, J., Guillermet, A. F., Grimvall, G., & Körling, M. Theory of bonding in transition-metal carbides and nitrides. *Phys. Rev. B* **48,** 11685 (1993).
